# Filamin-A-interacting protein 1 (FILIP1) is a dual compartment protein linking myofibrils and microtubules during myogenic differentiation and upon mechanical stress

**DOI:** 10.1007/s00441-023-03776-4

**Published:** 2023-05-13

**Authors:** Valentina Grande, Julia Schuld, Peter F. M. van der Ven, Oliver J. Gruss, Dieter O. Fürst

**Affiliations:** 1 Institute for Cell Biology, University of Bonn, Ulrich-Haberland-Str. 61a, 53121 Bonn, Germany; 2 Institute of Genetics, University of Bonn, Karlrobert-Kreiten-Str. 13, 53115 Bonn, Germany

**Keywords:** Filamin-A-interacting protein 1, Skeletal muscle differentiation, Myofibrillogenesis, Mechanical stress protection, Z-disc proteins

## Abstract

Variations in the gene encoding filamin-A-interacting protein 1 (*FILIP1*) were identified to be associated with a combination of neurological and muscular symptoms. While FILIP1 was shown to regulate motility of brain ventricular zone cells, a process important for corticogenesis, the function of the protein in muscle cells has been less well characterized. The expression of FILIP1 in regenerating muscle fibres predicted a role in early muscle differentiation. Here we analysed expression and localization of FILIP1 and its binding partners filamin-C (FLNc) and microtubule plus-end-binding protein EB3 in differentiating cultured myotubes and adult skeletal muscle. Prior to the development of cross-striated myofibrils, FILIP1 is associated with microtubules and colocalizes with EB3. During further myofibril maturation its localization changes, and FILIP1 localizes to myofibrillar Z-discs together with the actin-binding protein FLNc. Forced contractions of myotubes by electrical pulse stimulation (EPS) induce focal disruptions in myofibrils and translocation of both proteins from Z-discs to these lesions, suggesting a role in induction and/or repair of these structures. The immediate proximity of tyrosylated, dynamic microtubules and EB3 to lesions implies that also these play a role in these processes. This implication is supported by the fact that in nocodazole-treated myotubes that lack functional microtubules, the number of lesions induced by EPS is significantly reduced. In summary, we here show that FILIP1 is a cytolinker protein that is associated with both microtubules and actin filaments, and might play a role in the assembly of myofibrils and their stabilization upon mechanical stress to protect them from damage.

## Introduction

Cross-striated muscle cells express a unique contractile apparatus, in which the molecular components are arranged in an impressive, crystalline order. A major challenge is to define the steps and regulatory events of this supramolecular assembly process. Several models have been proposed, with the premyofibril model being the most accredited one (Sanger et al. 2005). According to this model, the assembly process starts in the cortex of muscle cells with premyofibrils, formed from minisarcomeres bounded by α-actinin in Z-bodies that contain muscle actin and a nonmuscle isoform of myosin II. The alignment of premyofibrils, their growth in width and the incorporation of titin and muscle myosin II (in addition to nonmuscle myosin II), leads to the formation of nascent myofibrils. To finalize the development of mature myofibrils, Z-bodies gradually become Z-discs, nonmuscle myosin is no longer present, and myosin-binding proteins are incorporated into aligned A-bands with central M-bands (Sanger et al. 2005, 2010). An involvement of microtubules in these processes was suggested, but mechanistic investigations have remained relatively scarce (Antin et al. 1981; Holtzer et al. 1985; Pizon et al. 2002, 2005; Dhanyasi et al. 2021). The overall organization of microtubules changes considerably during myogenesis, from a radial orientation in myoblasts to a parallel array in myotubes (Warren 1974; Kano et al. 1991). The latter is thought to enable or facilitate the division of the cytoplasm into myofibril-containing areas and an organelle-rich compartment (Dhanyasi et al. 2021).

Once established, the integrity of the contractile machinery is constantly challenged by the mechanical stress imposed by muscular activity. Mechanical forces in the low picoNewton range, as exerted by single myosin molecules, are strong enough to induce the unfolding of, e.g. immunoglobulin (-like) domains (Rief et al. 1997, 2000; Rognoni et al. 2012, 2014). Such unfolding events may be utilized for mechanotransduction and strain/stress sensing in the contractile apparatus, particularly in Z-discs and M-bands, the two structures that interconnect thin and thick filaments, respectively. (Partial) protein unfolding does, however, hold the risk to result in aggregation, and an extensive machinery is required to monitor and manage such events (reviewed in Höhfeld et al. 2021). This is of immediate importance when excessive mechanical stress threatens to rupture a myofibril. Therefore, such focal weak points, called lesions, have to be stabilized and repaired immediately. In the microscope they are recognizable as focal disruptions of the cross-striated myofibrillar pattern (Fridén et al. 1981, 1983; Yu et al. 2002, 2003, 2013; Chevessier et al. 2015; Schuld et al. 2020), and immunofluorescence microscopy revealed the large actin-crosslinking protein filamin-C (FLNc) as an early and specific marker for these regions (Chevessier et al. 2015; Leber et al. 2016; Orfanos et al. 2016; Schuld et al. 2020). Its molecular architecture of 24 immunoglobulin-like domains that bind a plethora of different proteins, may on the one hand tolerate partial unfolding, on the other hand it may orchestrate sarcomere repair by recruiting signalling and structural components (Ehrlicher et al. 2011; Rognoni et al. 2012; Leber et al. 2016; Schuld et al. 2020).

Filamin-A-interacting protein 1 (FILIP1, introduced as FILIP) was originally identified in a circumscribed zone in the developing brain, where it is involved in the degradation of filamin-A (FLNa), resulting in a stop of the migration of neocortical cells (Nagano et al. 2002, 2004). More recently, it was also identified as a direct binding partner of FLNc (Reimann et al. 2020). Interestingly, the PI3K/Akt pathway that promotes muscle growth and differentiation, phosphorylates FLNc in a basophilic amino acid motif that is not present in FLNa. This phosphorylation prevents FILIP1 binding and thus FLNc degradation, while FLNa is selectively eliminated, providing a mechanism for the observed switch of filamin isoforms during muscle development (Reimann et al. 2020). Recently, FILIP1 has also been shown to bind RhoD (Gad et al. 2012), a member of the RhoGTPases family. RhoGTPases stabilize microtubules, regulate the actin cytoskeleton and support changes in cell-shape (Cook et al. 1998; Daub et al. 2001; Palazzo et al. 2001; Fukata et al. 2002; Hall 2005). In addition, FILIP1 regulates the actin-binding activity and subcellular organization of non-muscle myosin (Yagi et al. 2014, 2017).

All these findings imply a link of FILIP1 to the actin cytoskeleton, but also to microtubules. Interestingly, a proteome-wide screen identified FILIP1 as a novel protein capable of binding to plus ends of microtubules via an interaction of its SxIP amino acid motifs with end-binding proteins (EBs; (Jiang et al. 2012). EBs are part of the heterogeneous group of microtubule plus-end-tracking proteins (+TIPs) with a C-terminal EB homology domain that allows the localization of other +TIPs to microtubules (Honnappa et al. 2009; Kumar et al. 2017). The EB protein family is composed of three members: EB1 and 2 are ubiquitously expressed, whereas EB3 is more restricted to brain and muscle (Su and Qi 2001; Nakagawa et al. 2000). In muscle cells, EB3 has a fundamental role in myogenic differentiation and its knockdown prevents myoblast elongation and fusion into myotubes (Straube and Merdes 2007).

These findings place FILIP1 at the scantly investigated interface between microfilament and microtubule cytoskeletal structures. We therefore analysed its subcellular distribution in adult skeletal muscle tissue, and during myogenic differentiation and found a transition from a preferred localization at microtubule ends at early stages, to myofibrillar Z-discs upon differentiation. Furthermore, it translocates together with FLNc to sarcomeric lesions induced by mechanical stress, where it might mediate contact to dynamic microtubules.

## Materials and methods

### Cell culture

C2C12 cells were grown in Dulbecco’s modified Eagle medium (DMEM), with GlutaMax and high glucose, containing 15% foetal calf serum (FCS), 1% penicillin/streptomycin and 1% non-essential amino acids (all from Gibco/ThermoFisher, Dreieich, Germany). Differentiation into myotubes was achieved by replacing FCS with 2% horse serum (Gibco/ThermoFisher) when cells reached 90% confluence. Media were changed every two days. Since the precise timing of myotube differentiation may vary between cell batches, we grouped them according to their respective developmental stage: (1) proliferating myoblasts, (2) premyofibril stage, usually achieved after 1 to 2 days of differentiation, (3) nascent myofibril stage, usually achieved after 3 to 4 days, (4) mature myofibril stage, usually after 5 to 6 days. For immunoblot analyses, cells were seeded on standard 100 mm tissue culture dishes (Sarstedt, Nümbrecht, Germany). For immunofluorescence microscopy, cells were grown on 12 mm diameter glass coverslips placed in culture dishes. Electrical pulse stimulation (EPS) was performed in 6-well plates.

### Immunofluorescence microscopy

For immunostainings, cells grown on coverslips were fixed in a 1:1 mixture of methanol and acetone at -20 °C for 2 min. Samples were blocked in 10% normal goat serum (NGS) and 1% BSA in phosphate-buffered saline (PBS) for 1 h. After overnight incubation at 4 ºC with mixtures of primary antibodies diluted in 3% NGS, 1% BSA in PBS, coverslips were washed three times with PBS containing 0.05% Tween 20 and incubated with appropriate secondary antibodies conjugated to Cy5, Alexa Fluor 488, 405 or 594plus (Jackson ImmunoResearch, Ely, United Kingdom or ThermoFisher) for 1 h at room temperature. After 2 washes with PBS and one with distilled water, coverslips were mounted with FluoromountG mounting medium. Cells were observed and photographed using a Cell Observer SD Spinning Disk Confocal Microscope and images were processed using Zen 3.6 software (Carl Zeiss, Oberkochen, Germany). Tissue cryosections were fixed in acetone at -20 °C for 10 min and air dried. Sections were rehydrated in PBS, blocked for 45 min with 10% goat serum, and consecutively incubated overnight at 4 ºC with primary antibodies diluted in PBS. After washing with PBS, samples were incubated with the appropriate secondary antibodies in PBS for 2 h at 37 ºC. Specimens were washed, mounted as described above, and photographed using an LSM710 confocal laser-scanning microscope (Carl Zeiss).

### Cell lysates, SDS–polyacrylamide gel electrophoresis and western blot analysis

For comparative protein quantification, cells were lysed with preheated SDS sample buffer, incubated for 15 min at 65 ºC, sonicated and centrifuged at 13,000 rpm for 15 min. A quantitative analysis of a Coomassie-stained SDS–polyacrylamide gel was used to adjust the total protein concentration of the lysates. Identical amounts of total cellular proteins were separated by SDS–polyacrylamide gel electrophoresis (Laemmli 1970) and transferred electrophoretically onto nitrocellulose membranes. Membranes were blocked with Tris-buffered saline-Tween (TBST: 0.02 M Tris, 0.150 M NaCl, 0.05% Tween 20) containing 5% non-fat dried milk or 3% BSA, and incubated overnight at 4 ºC with specific primary antibodies diluted in Tris-buffered saline with 0.05% Tween 20 (TBST) or in 2.5% non-fat milk powder in TBST. After washing 3 × 5 min with TBST, membranes were incubated either with IRDye-680- or IRDye-800-conjugated secondary antibodies (LI-COR Biosciences, Bad Homburg, Germany) or with peroxidase-conjugated secondary antibodies (Jackson ImmunoResearch) and ECL Western Blotting Substrate (ThermoFisher/Pierce). Blots were analysed using a ChemiDoc MP Imaging System (Bio-Rad, Feldkirchen, Germany). Densitometric analysis was carried out using the Image Lab software (6.1.0; Bio-Rad) using glyceraldehyde-3-phosphate dehydrogenase (GAPDH) as loading control.

### Electrical pulse stimulation (EPS) and nocodazole treatment

C2C12 myotubes were electrically stimulated using a C-pace unit and 6-well dishes (Ion Optix, Milton, MA, USA) as previously described (Orfanos et al. 2016). For mechanoadaptation of the cells a mild stimulation (pulses of 10 V for 4 ms at a frequency of 0.5 Hz) was used for 4 to 12 h. Sarcomeric lesions were elicited by applying a twitch protocol (pulses of 10 V for 10 ms at a frequency of 1 Hz) for different time periods (0.5, 1, 2 or 4 h). Subsequently, cells were fixed for immunostaining or lysed for western blot analyses as described above.

To induce sarcomeric lesions in the absence of microtubules, C2C12 myotubes were incubated for 2 h in differentiation medium containing 4 μg/ml nocodazole (NZ) and then electrically stimulated for 1 h by applying the twitch protocol, while control cells were incubated and electrically stimulated with the corresponding amount of dimethylsulfoxid (DMSO). After electrical stimulation, cells were shortly rinsed with warm Dulbecco's PBS and fixed for immunostaining. For rescue experiments, the NZ-containing medium was replaced by differentiation medium without NZ for 2 h. The number of lesions per $\mu$m^2^ was quantified from fluorescence micrographs as described previously (Orfanos et al. 2016).

### Antibodies

Details about all primary and secondary antibodies used in this study and their applied dilutions are summarized in Tables 1 and 2.

**Table 1:** List of primary antibodies used in this work

***Name***	***Antigen***	***Type***	***Dilution***	***Origin***
**Antibodies used for immunofluorescence microscopy:**				
**RR90**	filamin-A/C	mouse mAb IgA	1:30—1:50	van der Ven et al*.* (2000)
**FILIP1**	FILIP1	rabbit pAb	1:50—1:150	Atlas antibodies
**EB3 (7)**	EB3	mouse mAb IgG1	1:20	Santa Cruz Biotechnology
**Acetylated tubulin (Lys40)**	acetylated alpha-tubulin	mouse mAb IgG1	1:1,000	Proteintech
**YL1/2**	tyrosylated alpha-tubulin	rat mAb IgG2a	1:50	Wehland et al. (1983)
**E7**	beta-tubulin	mouse mAb IgG1	1:20	DSHB
**T12**	titin (Z-disc)	mouse mAb IgG1	1:20	Fürst et al. (1988)
**Antibodies used for western blot analysis:**				
**FLNc d16-20**	filamin-C	rabbit pAb	1:10,000	Chevessier et al. (2015)
**FILIP1**	FILIP1	rabbit pAb	1:250	Reimann et al. (2020)
**TUBA1B (clone 6-11B-1)**	acetylated tubulin alpha 1b	mouse mAb IgG2b	1:2,000	BioLegend
**YL1/2**	tyrosylated alpha-tubulin	rat mAb IgG2a	1:500	Invitrogen
**EB3**	EB3	rabbit pAb	1:2,000	Proteintech
**GAPDH (clone 5C6)**	GAPDH	mouse mAb IgG1	1:1,000	Calbiochem

**Table 2:** List of secondary antibodies used in this work

***Name***	***Donor***	***Directed against***	***Dilution***	***Supplier***
**Antibodies used for immunofluorescence microscopy:**				
**GAMouse IgA Alexa Fluor 488**	goat	mouse IgA	1:30	Southern Biotechnology Associates
**GARabbit Alexa Fluor 594plus**	goat	rabbit Ig	1:300	ThermoFisher
**GAMouse IgG1 Cy5**	goat	mouse IgG1	1:100	Jackson ImmunoResearch
**GARat Alexa Fluor 405plus**	goat	rat Ig	1:150	ThermoFisher
**GARabbit Cy3**	goat	rabbit Ig	1:300	Jackson ImmunoResearch
**Antibodies used for Western blot analysis:**				
**GAMouse-HRP**	goat	mouse Ig	1:10,000	Jackson ImmunoResearch
**GARabbit-HRP**	goat	rabbit Ig	1:30,000	Jackson ImmunoResearch
**GARat-HRP**	goat	rat Ig	1:10,000	Jackson ImmunoResearch
**GARabbit IRDye800**	goat	rabbit Ig	1:10,000	LI-COR
**GAMouse IRDye680**	goat	mouse Ig	1:5,000	LI-COR

*HRP* horse radish peroxidase, *IRDye* infrared dye

### General data analysis and figure preparation

Statistical analysis was performed using GraphPad Prism version 9 (GraphPad Software, La Jolla, CA, USA). Data in Fig. 2b–b'''' are expressed as mean ± SD. In every individual experiment the highest relative FILIP1 expression level from day 0 to day 8 was set to 1.0. Data in Figs. 3, 5 and 6 are expressed as mean ± SEM and were compared with One-way ANOVA followed by Tukey’s post hoc test or Kruskal–Wallis test followed by the post hoc Dunn’s multiple comparisons test. The normality of the distributions was assessed using the Shapiro–Wilk test. Differences were considered significant when p < 0.05. Figures were prepared using CorelDRAW Graphics Suite 2019 or 2020 (Corel, Austin, TX, USA).

## Results

### FILIP1 localizes to the myofibrillar Z-disc region and in myofibrillar lesions in adult skeletal muscle fibres

**Fig. 1:**
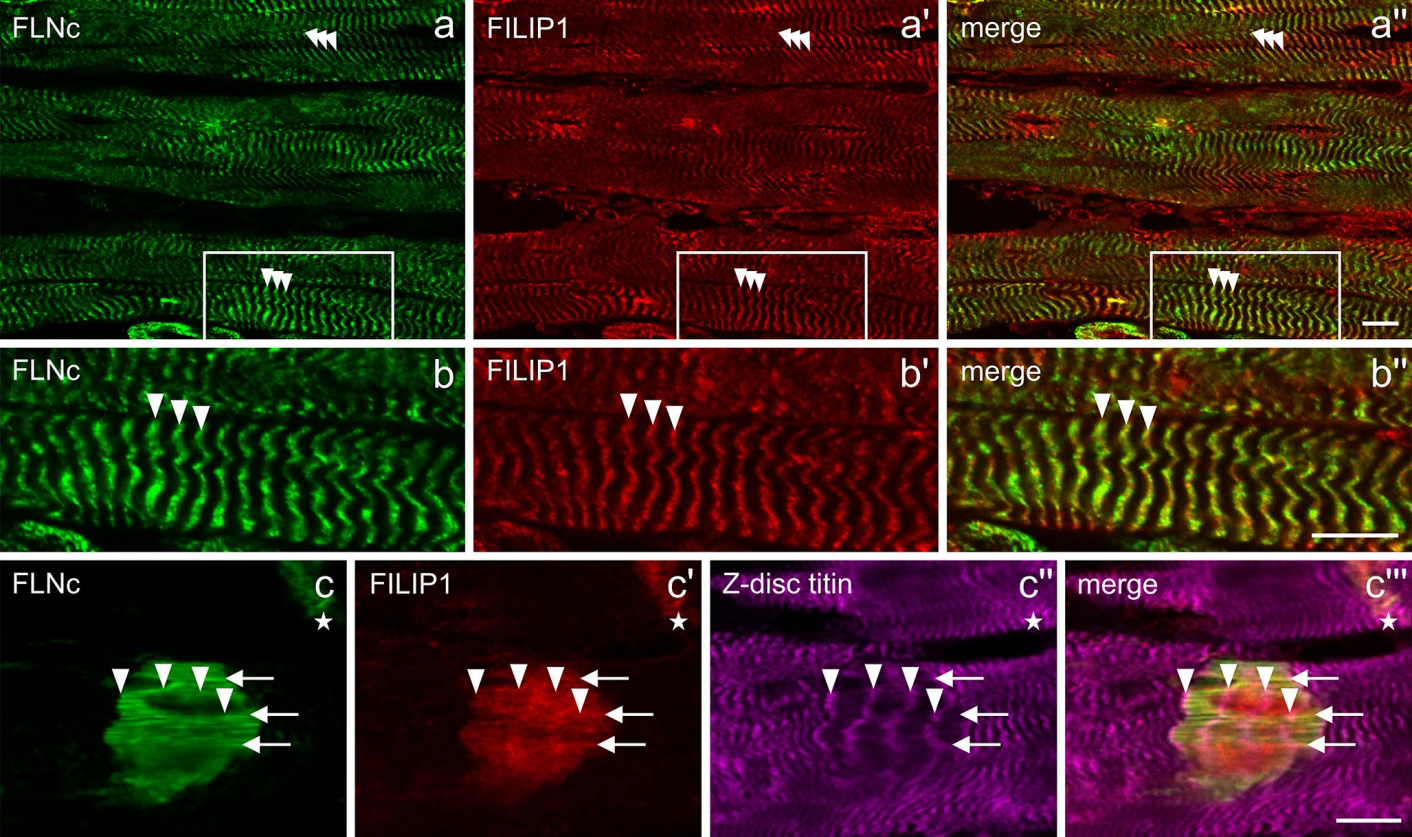
Localization of FILIP1 in adult mouse muscle. (**a**–**b''**) Immunolocalization of FLNc and FILIP1 in longitudinal cryosections of adult mouse soleus muscle indicates a localization of both proteins in the Z-disc region of the myofibrils (arrowheads). The boxed area in (**a**–**a''**) is shown enlarged in (**b**–**b''**). (**c**–**c'''**) Localization of FLNc, FILIP1 and a Z-disc epitope of titin in a macrolesion and a microlesion (asterisk). FLNc and FILIP1, but not titin, are concentrated in lesions. FLNc shows a more filamentous staining than FILIP1 that shows a more diffuse localization and is only associated with individual filaments (arrows). Arrowheads indicate the position of Z-discs within the macrolesion. Due to the short exposure because of the enhanced signal in the lesions, Z-disc staining of FLNc and FILIP1 is not visible. Bars: 10 m

Previously, we showed that FILIP1 not only binds FLNa, but also FLNc (Reimann et al. 2020). To compare the localization of both proteins in adult skeletal muscle, longitudinal cryosections of mouse soleus muscle were stained with a combination of antibodies specific for both proteins. FILIP1 was found to localize in a typical cross-striated pattern together with FLNc (Fig. 1a–a'', b–b''), indicating that FILIP1 is a novel myofibrillar Z-disc-associated protein. Furthermore, we found FILIP1 to be highly concentrated in myofibrillar lesions. Interestingly, in these structures FILIP1 was distributed more diffusely than FLNc and a Z-disc epitope of titin (Fig. 1c–c''').

### FILIP1 expression and localization in proliferating and differentiating C2C12 cells

**Fig. 2:**
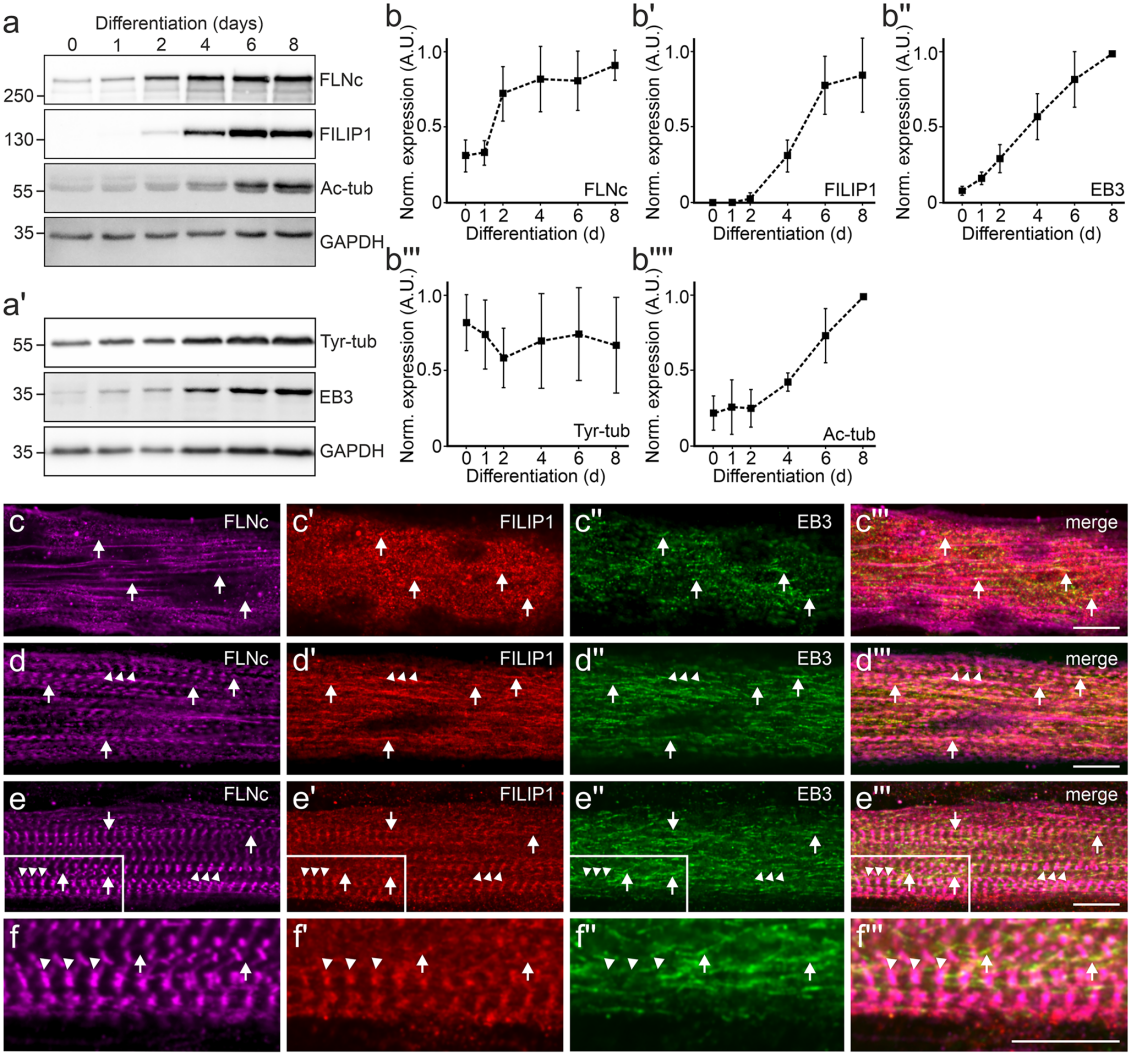
FILIP1 expression and localization in differentiating cultured C2C12 myotubes. **a**, **a'** Representative western blots showing the levels of FLNc, FILIP1, EB3, tyrosylated tubulin (Tyr-tub) and acetylated tubulin (Ac-tub) in proliferating C2C12 myoblasts (0 days) and myotubes differentiated for the indicated time (1–8 days). GAPDH was used as loading control and for normalization. **b**–**b''''** Quantification of the data presented in (**a**–**a'**), expressed as mean ± SD. n = 3. In all individual experiments maximum relative expression of each protein was set to 1.0. **c**–**e'''** Immunolocalization of FLNc, FILIP1 and EB3 in C2C12 myotubes differentiated for 2 (**c**–**c'''**), 4 (**d**–**d'''**) and 6 (**e**–**e'''**) days. **c**–**c'''** FILIP1 and EB3 partially colocalize (arrows), which becomes more evident later in differentiation (**d**–**d'''**, arrows). This is still evident in mature myotubes 6 days after the start of differentiation (**e**–**e'''**, arrows), when FILIP1 and FLNc are mainly present in Z-discs (**e**–**e'''** and **f**–**f'''**, arrowheads). A magnification of the boxed area in (**e**–**e'''**) is shown in (**f**–**f'''**). Bars: 10 μm

The recent discovery of EB1 and EB3 as FILIP1 binding partner (Jiang et al. 2012) warranted a careful analysis of the comparative expression and localization of these proteins together with FLNc in developing cultured muscle cells. Our western blot experiments show a steep increase of FLNc and EB3 expression already at the onset of myotube differentiation, while FILIP1 was detected only after two days of differentiation (Fig. 2a–a', b–b'').

At the premyofibril stage, the FILIP1 expression level is relatively low and a great proportion of the protein is diffusely localized (Fig. 2c'). At this time point, a defined partial co-localization is only apparent with EB3 but not with FLNc (Fig. 2c'–c''', arrows). With the appearance of nascent myofibrils, the localization of FILIP1 becomes more defined and still coincides solely with the localization of EB3 (Fig. 2d–d''', arrows). At this stage, FILIP1 is not associated with FLNc in Z-discs (Fig. 2d–d''', arrowheads). As mature myofibrils appear, FILIP1 localization changes considerably: the protein is primarily redistributed to Z-discs, where it colocalizes with FLNc (Fig. 2e–e''', f–f''', arrowheads). Co-localization with EB3 is still visible, but far less prominent (Fig. 2e–e''', f–f''', arrows).

Next, we investigated whether this conspicuous redistribution of FILIP1 correlated with specific post-translational modifications of tubulin. Western blotting experiments demonstrated that during myotube differentiation the level of acetylated tubulin, a marker of stable microtubules, increases considerably, especially after 2 and more days of differentiation. In contrast, the levels of tyrosylated alpha-tubulin, a marker of more dynamic microtubules, remained similar during the entire differentiation process (Fig. 2a–a', b'''–b'''').

### FILIP1 increasingly translocates and colocalizes with FLNc in EPS-induced sarcomeric lesions

**Fig. 3:**
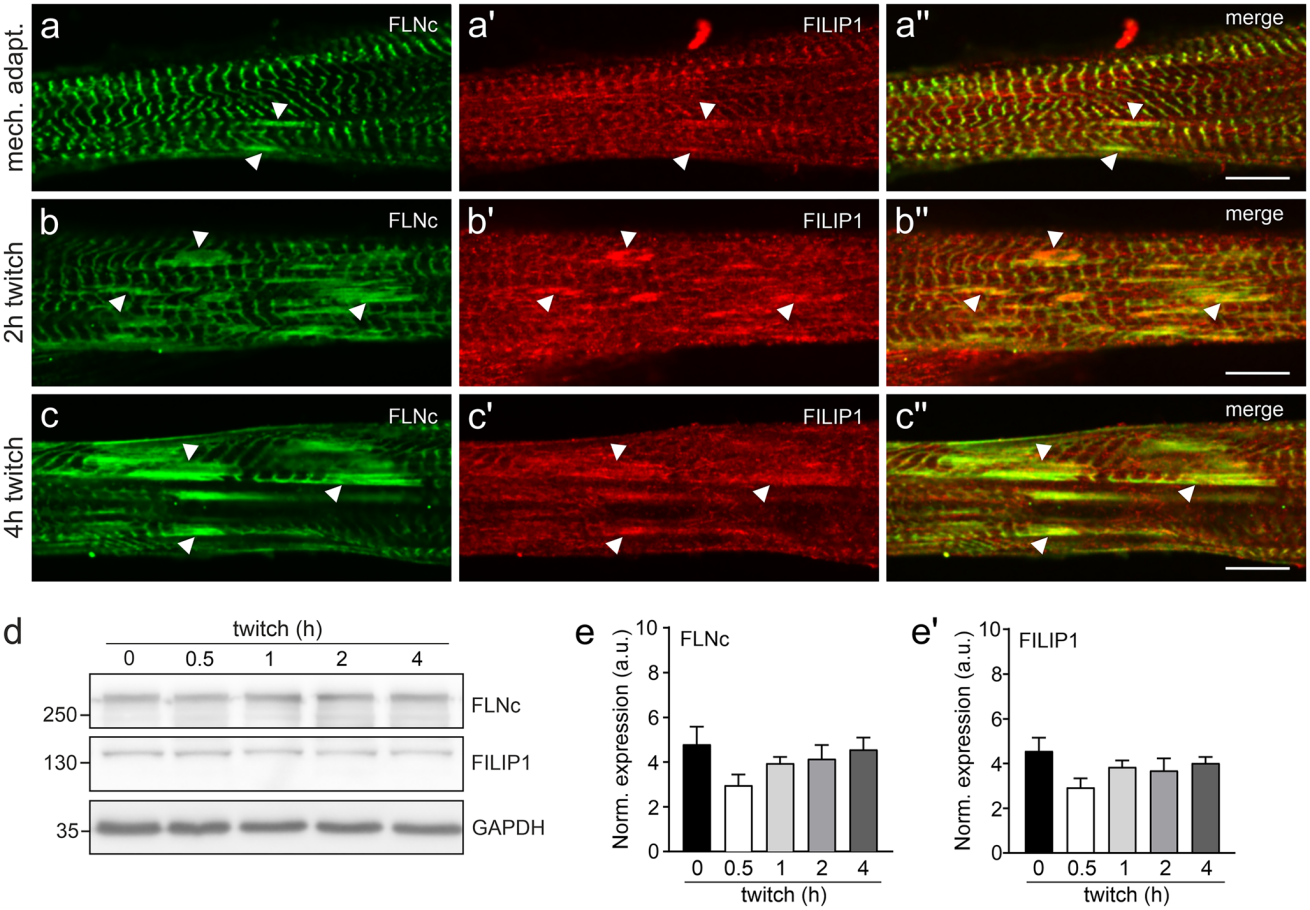
FILIP1 is localized in EPS-induced sarcomeric lesions. **a**–**a''** Immunolocalization of FLNc and FILIP1 in overnight mechanoadapted C2C12 myotubes (**a**–**a''**), followed by twitch stimulation for 2 h (**b**–**b''**) or 4 h (**c**–**c''**). Arrowheads indicate the presence of both FLNc and FILIP1 in EPS-induced lesions. Note that incidence and size of lesions as well as the presence of FILIP1 in the lesions increase with the intensity of the applied stimulus and duration of pacing. Bars: 10 μm. **d** Representative western blots showing the levels of FLNc and FILIP1 in mechanoadapted C2C12 myotubes treated with EPS for the indicated time periods. GAPDH was used as loading and normalization control. **e**–**e'** Quantification of the data shown in (**d**) expressed as mean ± S.E.M. Significance was assessed using One-way ANOVA and Tukey’s post hoc test or Kruskal–Wallis test and the post hoc Dunn’s multiple comparisons test. No significant differences were found

**Fig. 4:**
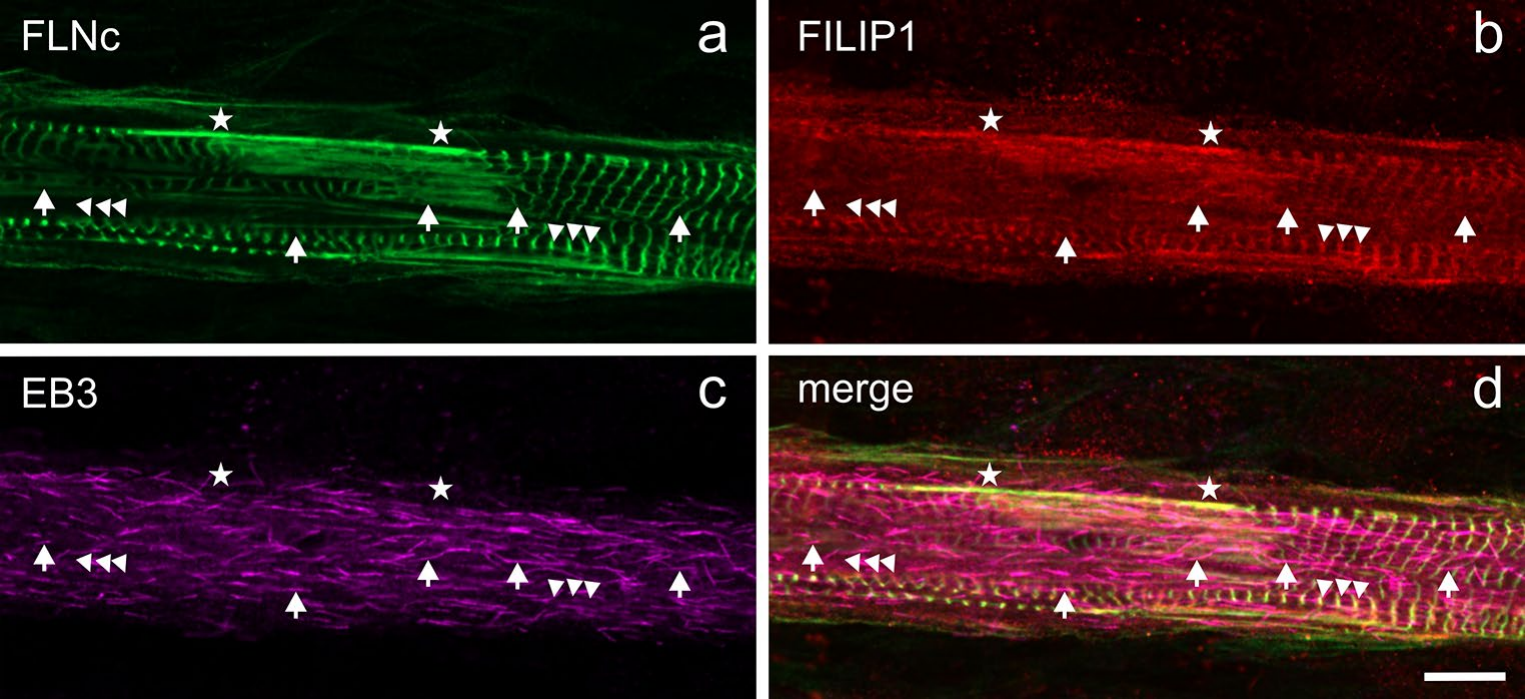
FILIP1 mainly colocalizes with FLNc but not with EB3 after EPS-treatment. Immunolocalization of FLNc (**a**), FILIP1 (**b**) and EB3 (**c**) in EPS-treated myotubes reveals the presence of both, FLNc and FILIP1 in the remaining Z-discs (arrowheads) and in lesions (asterisks), while FILIP1 does not colocalize with EB3 (arrows). The merged picture is shown in panel **d**. Bar: 10 μm

Previous investigations have demonstrated that FLNc and some of its binding partners are involved in managing sarcomeric lesion formation and repair (Orfanos et al. 2016; Leber et al. 2016; Schuld et al. 2020). The identification of FILIP1 as FLNc binding partner (Reimann et al. 2020) warranted to study its potential role in these processes. We therefore performed double immunofluorescence microscopy for FLNc and FILIP1 in C2C12 myotubes after twitch-induced sarcomeric lesion formation. Overnight mechanoadaptation using mild EPS (10 V, 4 ms, 0.5 Hz) induced a small amount of microlesions, spanning only a few sarcomeres identified by staining for FLNc (Fig. 3a–a''). In contrast, subsequent application of the more intense twitch protocol (10 V, 10 ms, 1 Hz) resulted in formation of macrolesions (lesions involving more than 5 sarcomeres and across multiple myofibrils), increasing in dimension over time (Fig. 3b–b'', c–c''). After mechanoadaptation, FILIP1 predominantly showed a Z-disc staining pattern, while lesions were less intensely stained (Fig. 3a–a''). Twitch-induced mechanical stress subsequently resulted in a striking redistribution of FILIP1 from Z-discs to lesions, with a concomitant increase upon longer stimulation (Fig. 3b–b'', c–c''). To analyse whether this redistribution is associated with an increased expression of FILIP1, we quantified the protein levels of FLNc and FILIP1 by western blotting. This revealed a small, but non-significant transient decrease of FLNc and FILIP1 protein amounts, and stable protein levels until the end of the experiment (Fig. 3d, e–e'). These findings corroborate our assumption, based on immunofluorescence microscopy data, that upon mechanical stress, both FLNc and FILIP1 rapidly translocate from their initial localization in Z-discs to lesions. To investigate whether FILIP1 localization in EPS-treated myotubes reverts to its early developmental localization pattern in proximity to EB3, we triple stained such myotubes for FLNc, FILIP1 and EB3. The FILIP1 signal mainly remained together with FLNc in Z-discs and myofibrillar lesions. An increase in colocalization with EB3 was not observed (Fig. 4a–d).

### Tyrosylated microtubules flank sarcomeric lesions induced by EPS

**Fig. 5:**
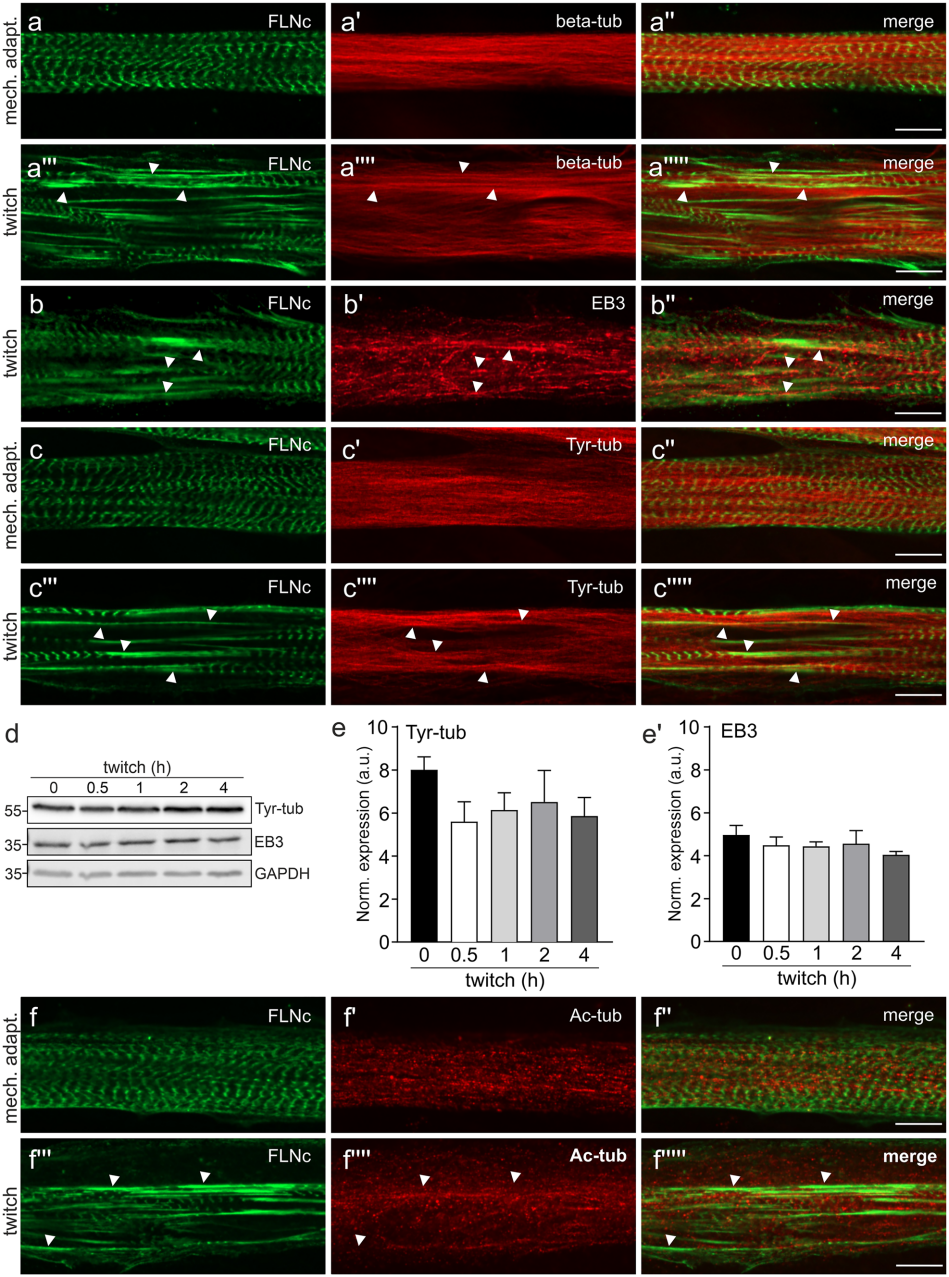
Tyrosylated microtubules flank EPS-induced sarcomeric lesions. **a–a'''''** Microtubules stained for beta-tubulin show the typical longitudinal orientation in mechanoadapted (mech. adapt.) myotubes (**a**–**a''**) while in twitch-paced myotubes many sarcomeric lesions are flanked by microtubules (**a'''**–**a'''''**, arrowheads). **b**–**b''** Immunolocalization of FLNc and EB3 in a C2C12 myotube treated with twitch EPS. The arrowheads indicate sarcomeric lesions marked by FLNc. EB3 is widely expressed throughout the myotube, and appears concentrated in the proximity of these lesions. **c**–**c''** Immunolocalization of FLNc and tyrosylated tubulin (Tyr-tub) in C2C12 myotubes treated with basic EPS (**c**–**c''**) and twitch EPS (**c'''**–**c'''''**). Arrowheads indicate sarcomeric lesions, strongly positive for FLNc, which are flanked by tyrosylated microtubules. **d** Representative western blots showing the expression level of tyrosylated tubulin (Tyr-tub) and EB3 in mechanoadapted C2C12 myotubes that were twitch EPS treated for the indicated time periods. GAPDH was used as loading and normalization control. **e**–**e'** Quantification of the data shown in (**d**) expressed as mean ± S.E.M. n = 3. Significance was assessed using One-way ANOVA and Tukey’s post hoc test or Kruskal–Wallis test and the post hoc Dunn’s multiple comparisons test. No significant differences were found. **f**–**f'''''** Immunolocalization of FLNc and acetylated tubulin in C2C12 upon mechanoadaptation (**f**–**f''**) or twitch EPS (**f'''**–**f'''''**). Acetylated tubulin is not particularly arranged in close proximity to lesions (**f'''**–**f'''''**, arrowheads). Bars: 10 μm

The microtubule cytoskeleton, and in particular certain tubulin post-translational modifications, seem to play an important role in striated muscle function (Kerr et al. 2015; Robison et al. 2016; Sébastien et al. 2018). Since FILIP1 transiently colocalizes with EB3 during early stages of myofibril formation (see Fig. 2), we next investigated the localization of its binding partner FLNc in relation to microtubules and EB3 in mechanoadapted and twitch EPS-treated myotubes, particularly with respect to increasing lesion formation. In mechanoadapted myotubes, microtubules showed the typical longitudinal orientation, while FLNc was localized in Z-discs (Fig. 5a–a''). Upon twitch EPS, we found microtubules (Fig. 5a'''–a''''') and EB3 (Fig. 5b–b'') to be more accentuated in immediate proximity to sarcomeric lesions. EB3 is a marker for growing microtubule ends, and thus would be predicted to colocalize with tyrosylated tubulin ('Tyr-tubulin'), which predominantly occurs in newly formed microtubules. Our immunolocalization studies revealed that Tyr-tubulin is located in a loosely arranged network surrounding the myofibrils, predominantly along the long axis of the myotubes (Fig. 5c–c''). In twitch EPS-treated myotubes, this pattern is more intense in regions flanking sarcomeric lesions (Fig. 5c'''–c'''''). These microscopical analyses were complemented by western blots using antibodies against Tyr-tubulin and EB3 (Fig. 5d). Quantitative evaluation, using GAPDH as a standard, did not reveal significant EPS-induced changes in the expression levels of EB3 or tyrosylated tubulin (Fig. 5e–e'). Staining with antibodies detecting acetylated tubulin did not reveal a distinct co-localization of acetylated microtubules in relation to Z-discs (Fig. 5f–f'') or sarcomeric lesions (Fig. 5f'''–f''''').

### Microtubule network depolymerization by nocodazole does not affect the localization of FILIP1 at the Z-disc and its presence in sarcomeric lesions

**Fig. 6:**
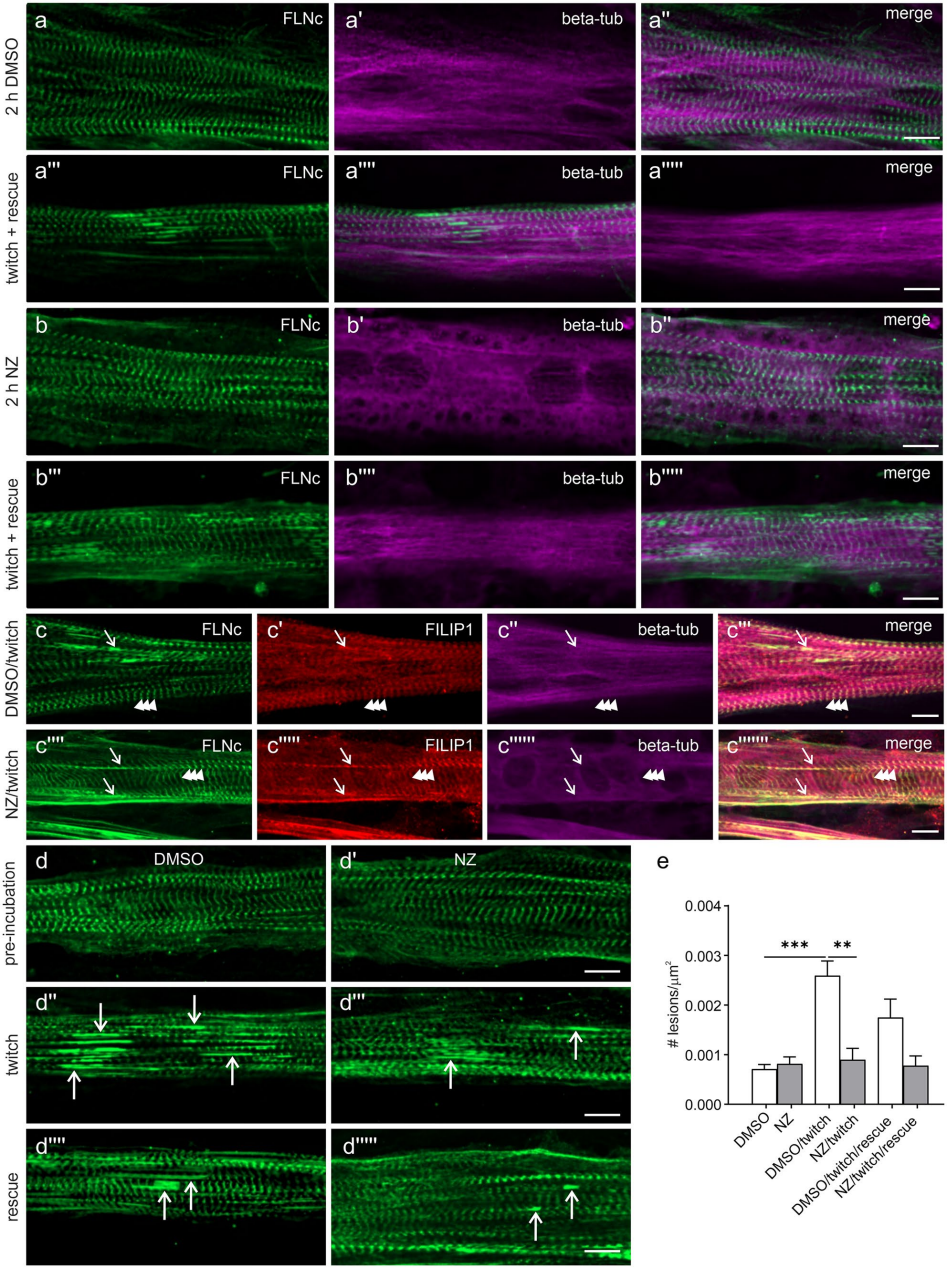
Microtubule depolymerization does not affect the localization of FILIP1 at Z-discs and in sarcomeric lesions. **a**–**a'''''**, **b**–**b'''''** Immunolocalization of FLNc and total tubulin (beta-tub) in C2C12 myotubes treated with (**a**–**a'''''**) DMSO or (**b**–**b'''''**) 4 μg/ml nocodazole (NZ). NZ treatment for 2 h induced complete depolymerization of the microtubule network, which was rescued within 2 h after washout of NZ, after the EPS treatment. **c**–**c'''''''** Representative images of C2C12 myotubes treated with EPS and DMSO or NZ and stained for FLNc, FILIP1 and total tubulin (beta-tub). The localization of FILIP1 at the Z-discs (indicated by arrowheads) and in sarcomeric lesions (indicated by arrows) is not affected by the treatment with NZ. **d**–**d'''''** Representative images of C2C12 myotubes pre-incubated for 2 h with DMSO (**d**) or NZ (**d'**), subsequently treated with twitch EPS for 1 h to induce sarcomeric lesions (**d''**–**d'''**), and rescued by a 2 h incubation with medium without DMSO or NZ (**d''''**–**d'''''**). Myotubes were stained for FLNc. The arrows indicate EPS-induced lesions. Note that treatment with NZ seems to reduce the number of lesions induced by EPS. **e** Histogram showing the number of sarcomeric lesions (expressed as # of lesions/$\mu$m^2^) induced by the indicated different experimental conditions used in (**d**–**d'''''**). Twitch EPS significantly increased the number of lesions, but not in myotubes treated with NZ. Data are expressed as mean ± S.E.M. 9–18 cells were analysed for each experimental condition. Significance was assessed using the Kruskal–Wallis test and the post hoc Dunn's multiple comparisons test. **p < 0.01; ***p < 0.001

FILIP1 can interact with both actin-binding, as well as microtubule-binding proteins. Therefore, we investigated whether perturbation of microtubules by the application of the drug nocodazole (NZ) impacts the Z-disc localization of FILIP1 and its mechanical stress-dependent redistribution to sarcomeric lesions. To induce a complete depolymerization of the microtubule network, after mechanoadaptation, cells were pre-incubated for 2 h in medium containing 4 μg/ml NZ, and then twitch-paced for 1 h to induce the formation of sarcomeric lesions. Subsequently, cells were either fixed or allowed to recover for 2 h in medium without NZ, allowing the reformation of microtubules and an analysis of a possible effect on the number of sarcomeric lesions. Control cells incubated with only the solvent of NZ (DMSO) do not show any effect on the microtubule network, irrespective of EPS treatment (Fig. 6a–a'''''). In contrast, the microtubule-depolymerizing effect of NZ is clearly evident (Fig. 6b–b'''''). The localization of both FLNc and FILIP1 in Z-discs and sarcomeric lesions was not affected by microtubule depolymerization (Fig. 6c–c'''''). A detailed comparison of the NZ-treated and control samples indicated that NZ-treated myotubes contain less myofibrillar lesions (Fig. 6d–d'''''). Quantification of the total number of lesions in NZ-treated myotubes and untreated cells revealed that after mechanoadaptation these values were not significantly different (Fig. 6e). Subsequent twitch pacing, however, triggered the formation of a significantly larger number of lesions in control myotubes when compared to NZ-treated cells lacking intact microtubules (Fig. 6e). During the recovery period after removal of NZ, the number of lesions was reduced in control myotubes, while in NZ-treated myotubes the number remained at the original low level (Fig. 6e). In summary, the number of sarcomeric lesions was significantly affected by the presence or absence of microtubules during twitch pacing.

## Discussion

FILIP1 was originally described as "filamin-A-interacting protein" in brain ventricular zone cells. Here, it was shown to induce the degradation of FLNa, thereby terminating the migration of neocortical cells from the ventricular zone (Nagano et al. 2002, 2004). The abundant expression of FILIP1 in developing striated muscle cells and the dominant negative effect on myogenic differentiation by FILIP1 knockdown suggested an important role also in these cells (Militello et al. 2018; Reimann et al. 2020). In a previous study, in which we analysed PI3K/Akt signalling in developing muscle cells, we found a phosphorylation-dependent differential role of FILIP1 in FLNa degradation versus FLNc stabilization, which has important implications for distinct roles of filamin variants during muscle differentiation (Reimann et al. 2020). The role of FILIP1 in *developing* muscle cells may therefore be, similar to its proposed function in neuronal cells, to cease the migration of myoblasts after their migration to the final destination and prior to the terminal differentiation into myotubes by reducing FLNa levels (Nagano et al. 2002).

This assumption leaves open the function of FILIP1 at its Z-disc localization in adult muscle fibres (Fig. 1) and in terminally differentiated cultured myotubes, in which expression levels continue to be high (see Fig. 2 and (Reimann et al. 2020). Here, a surprising but important finding was the identification of amino acid sequence motifs (SxIP), that probably mediate binding to the microtubule plus-end tracking proteins EB1 and EB3 (Jiang et al. 2012). This raised the question whether FILIP1 might function as an adapter protein that is involved in a crosstalk between the actin cytoskeleton and microtubules. In this respect it is interesting to note that both, EB1 and EB3 are important for microtubule organization in myocytes (Straube and Merdes 2007; Zhang et al. 2009). Our western blots clearly show a synchronous and strongly increasing expression of FLNc, FILIP1 and EB3 upon myogenic differentiation (Fig. 2). Previous investigations already demonstrated that EB3 is the most prevalent EB family member in brain and muscle (Nakagawa et al. 2000; Su and Qi 2001), and that its expression level increases during myogenic differentiation (Straube and Merdes 2007). This prompted us to investigate two important posttranslational modifications of tubulin that have functional implications, namely acetylation and tyrosylation: while tyrosylation preferentially occurs on younger and more dynamic microtubules, acetylation is considered to stabilize microtubules and to counteract mechanical strain (Eshun-Wilson et al. 2019; Robison et al. 2016). Our immunoblots clearly revealed increasing tubulin acetylation relative to tyrosylation during differentiation, implying microtubule stabilization in response to the onset of contractile activity (Fig. 2a–a', b'''–b''''). Such changes in cytoskeletal stiffness were shown to allow for better transmission of mechanical signals (Kerr et al. 2015; Coleman et al. 2021) and to improve muscle repair and maintenance (Moris et al. 2017). In contrast, deregulation of these tubulin modifications was found in diseased muscles and overexpression of tubulin tyrosine ligase was used to attenuate contraction-induced injuries in a Duchenne muscular dystrophy mouse model and to improve the contractile kinetics in cardiomyocytes (Kerr et al. 2015; Chen et al. 2018).

To reveal possible spatial connections of FILIP1 with FLNc and EB3, we investigated their relative localization at high resolution in differentiating muscle cells using confocal immunofluorescence microscopy. Here we observed a striking transition from a colocalization of FILIP1 mainly with EB3, i.e. microtubule plus ends, to a preferred colocalization with FLNc in Z-discs (Fig. 2c–f'''), implying FILIP1 as an important adapter protein between sarcomeres and the intermyofibrillar cytoskeleton. Unlike in most other cell types, microtubules themselves undergo a drastic shape change during myogenic differentiation, from a network to mainly bundles running in parallel to the long axis of developing myofibrils. This setup was proposed to help to transport and organize myosin into A-bands in conjunction with MuRF family E3 ligases (Pizon et al. 2005). Further stabilization of this arrangement is achieved by tubulin acetylation, which has implications for mechanotransduction and mechanical resistance to contraction-induced damage, particularly in disease states (Kerr et al. 2015; Coleman et al. 2021; Chen et al. 2018). Focal, mechanical force-induced myofibrillar damage is, however, a constant threat to the integrity of the contractile apparatus and efficient repair mechanisms are therefore essential (Fridén et al. 1981, 1983; Yu et al. 2003, 2002, 2013; Orfanos et al. 2016; Leber et al. 2016). To address in this situation the interplay between microtubules, FILIP1 and FLNc, we used our established cell model of C2C12 myotubes, in which graded damage is induced by different regimes of electrical pulse stimulation [EPS; (Orfanos et al. 2016; Molt et al. 2014; Lohanadan et al. 2021)]. Already at relatively mild EPS conditions focal disruptions become evident, which may easily be visualized by using antibodies against filamin (see Fig. 3a–a''). Upon continued forced contractions their dimensions increase with time (see Fig. 3b–b'', c–c''). Clearly, FILIP1 follows this intensified staining of FLNc in lesions relative to Z-discs (Fig. 3a–a''). It is important to note that the total amounts of both proteins did not change significantly during the entire experiment (see Fig. 3d, e–e'), implying that we observe a translocation rather than incorporation of newly synthesized protein. FLNc, which is able to translocate away from Z-discs within seconds (Leber et al. 2016), may thus serve both as a mechanical stress sensor (Ehrlicher et al. 2011; Rognoni et al. 2012, 2014; Ulbricht et al. 2013) and as a docking platform for additional components. An essential process in this context is packaging of mechanically damaged proteins into autophagic vesicles, which involves the FLNc-binding SYNPO2 as an adapter protein to the "chaperone-assisted selective autophagy" (CASA) machinery (Ulbricht et al. 2013, Arndt et al. 2010, Lohanadan et al. 2021, reviewed in Höhfeld et al. 2021). Subsequently, these vesicles have to be transported to lysosomes, which requires intact microtubules and motor proteins of the dynein class (see e.g. Magupalli et al. 2020; Rubinsztein et al. 2005; Cabukusta and Neefjes 2018; Pu et al. 2016; Saftig and Klumperman 2009). Here, FILIP1 may be important to mediate linkage of myofibrillar lesions to the microtubular system via its interaction with EB1 and EB3 (Jiang et al. 2012). In this scenario, we may even assume a synergistic role of FILIP1 and MuRF family proteins. The latter associate with microtubules, myosin and titin during sarcomere formation, and may associate with the M-band in mature myofibrils (Pizon et al. 2002). In addition, MuRF1 and MuRF2 seem to interact with a shared set of proteins that include the Z-disc proteins FLNc and myotilin (Witt et al. 2008). Taken together, these findings suggest a synergistic role of FILIP1 and MuRFs, integrated by FLNc, in controlling myofibril repair. Constituting tubulin subunits of microtubules, in turn, undergo multiple posttranslational modifications, thereby influencing microtubule dynamics. In short, tyrosylation is taken as a sign for a more dynamic subpopulation, while acetylation is more prevalent in more stable, long-lived microtubules (reviewed in Wloga et al. 2017). Our immunofluorescence analyses now reveal tyrosylated microtubules flanking myofibrillar lesions, implying increased microtubule dynamics in these areas (see Fig. 5), which might relate to locally induced CASA activity. While such findings might be applied to implicate that certain drugs influencing microtubule dynamics were beneficial in disease states, such arguments have to be taken with great caution. Generally, diseased cardiac and skeletal muscle cells display significant changes in tubulin posttranslational modifications, concomitant with increased ROS production (Coleman et al. 2021). Indeed, an overexpression of the enzyme tubulin tyrosine ligase resulted in both reduced ROS levels and reduced contraction-induced damage in mdx-mice (Kerr et al. 2015). Likewise, suppression of detyrosylated microtubules was shown to improve cardiomyocyte function in human heart failure (Chen et al. 2018). In contrast, a drug-based approach to destroy the microtubule network in vivo, improved muscular damage in mdx-mice (Khairallah et al. 2012). In line with this, our EPS-stimulated muscle cells with depolymerized microtubules displayed less contraction-induced damage, visible as FLNc containing lesions with associated FILIP1, while at the same time the association of FLNc and FILIP1 with Z-discs and lesions was unaffected (see Fig. 6).

Interestingly, FILIP1 was recently identified as a potential disease gene associated with neurological and muscular manifestations (Gulsuner et al. 2013; Al-Kasbi et al. 2022), underlining its important role in both tissues. Although further investigations are needed to unravel the precise role of FILIP1, this disease association and its connection to microtubules and the actin cytoskeleton indicate a substantial function for the protein, not only in myofibril assembly and sarcomeric damage repair, but also in nervous tissue.
